# Assessing knowledge and lifestyle behaviours for hypertension management among adults in urban Ghana: a cross-sectional study

**DOI:** 10.1017/S1463423626101133

**Published:** 2026-05-06

**Authors:** Theodora Ojangba, Solomon Boamah, Yudong Miao, Shen Zhanlei, Richard Dormatey, Dongfang Zhu, Robert Dedi, Wenyong Dong, Qiuping Zhao, Baoyong Hua

**Affiliations:** 1 College of Public Health, Zhengzhou University, Zhengzhou, Henan, China; 2 Ghana Health Service, Juaboso District Health Directorate, Ghana; 3 Crops Research Institute (CSIR), Kumasi, Ghana; 4 Ghana Health Service, Adaklu District Health Directorate, Ghana; 5 Department of Hypertension, Henan Provincial People’s Hospital, People’s Hospital of Zhengzhou University, Henan, China; 6 Henan Key Laboratory for Health Management of Chronic Diseases, Central China Fuwai Hospital, Central China Fuwai Hospital of Zhengzhou University, Zhengzhou, Henan, China

**Keywords:** adherence, Africa, cardiovascular disease, Ghana, health management, high blood pressure, knowledge, lifestyle modification, nutrition, risk factors

## Abstract

**Aim:**

This study aimed to assess knowledge, lifestyle behaviours, and sociodemographic associations regarding hypertension control among adults in urban Ghana.

**Background:**

Hypertension is a major contributor to cardiovascular morbidity and mortality in Ghana. However, data on population-level knowledge of its risk factors and related lifestyle behaviours in urban settings remain limited.

**Methods:**

A cross-sectional analytical survey was conducted between August 2023 and September 2024 across four urban regions. Using stratified convenience sampling, 7096 adults aged 18–67^+^ years were recruited. Data on sociodemographic, lifestyle behaviours, and hypertension knowledge were collected via a structured questionnaire.

**Findings:**

Participants had a mean age of 37.27 (±8.73) years, with a majority being female (63.85%) and married (97.66%). Educational attainment varied. Females constituted most hypertensive cases, particularly for stage 2 hypertension, while males had a notably higher prevalence of pre-hypertension among those aged 27–53 years. Age and body mass index showed significant positive correlations with systolic and diastolic blood pressure (*p* < 0.01). Men were significantly more likely to smoke and consume alcohol (*p* < 0.01). Logistic regression indicated that regular exercise reduced the odds of hypertension diagnosis (OR = 0.72, CI: 0.54–0.96), while older age increased the odds. The study underscores the need for targeted public health strategies. Priorities include promoting physical activity and weight management, alongside smoking/alcohol cessation programs tailored for high-risk men. Early intervention for younger adults with pre-hypertension and enhanced educational outreach for less-educated groups are crucial.

## Background

Hypertension (HTN) is the most prevalent cardiovascular disease among adults and is widely recognized as a major premorbid factor linked to numerous chronic disease risk factors (Cimmaruta *et al*., 2018). The Global Burden of Disease study identified hypertension as a leading contributor to disability-adjusted life years worldwide (Abegaz *et al*., 2017). According to the World Health Organization (WHO), approximately 1.13 billion people globally are affected by hypertension, around 20% of women and 24% of men (Riaz *et al*., 2023). In 2015, the highest prevalence of arterial hypertension (AH) among men was reported in Croatia, Latvia, Lithuania, Hungary, and Slovenia, while the highest rates among women were found in Niger, Chad, Mali, Burkina Faso, and Somalia, where nearly 30% of the population was affected (Osmanović *et al.*, 2022).

According to the WHO, hypertension is defined as a cardiovascular condition characterized by a sustained systolic blood pressure (SBP) of 140 mmHg or higher and/or a diastolic blood pressure (DBP) of 90 mmHg or higher (Carretero and Oparil, 2000). A 20/10 mmHg increase in blood pressure is associated with a twofold higher risk of cardiovascular events in certain age groups. The primary goal of antihypertensive therapy is to achieve optimal blood pressure levels and reduce the risk of cardiovascular complications (Saiz *et al*., 2022). In addition to medication, non-pharmacological interventions such as lifestyle modifications, a healthy diet, regular physical activity, reduced alcohol intake, and smoking cessation are also recommended (Kodela *et al*., 2023). Adherence is essential for the effectiveness of antihypertensive therapy and successful blood pressure (BP) control (Peacock and Krousel-Wood, 2017). However, accurately assessing adherence remains a challenge for healthcare professionals. Various approaches, such as monitoring lifestyle modifications, conducting pill counts, evaluating clinical outcomes, and utilizing electronic medication monitoring systems, have been employed to measure adherence (Schnorrerova *et al*., 2024). Multiple factors influence a patient’s adherence, including the type and severity of the disease, individual patient characteristics, socioeconomic status, treatment type and regimen, as well as healthcare provider-related factors (Peh *et al*., 2021).

Knowledge plays a key role in enhancing both confidence and adherence to treatment regimens of hypertension (Akoko *et al*., 2017). Individuals who understand the relationship between diet and HTN are more likely to adopt healthier eating habits with greater confidence. According to the outcome dimension of the Individual and Family Self-Management Theory (IFSMT), healthy dietary and lifestyle changes can promote motivation and improve behavioural change self-efficacy, ultimately supporting better adherence to treatment (Angraini and Febriani, 2024; Li *et al*., 2024). Additionally, the context dimension of IFSMT suggests that the physical and psychological well-being of individuals with hypertension can influence their self-efficacy and adherence. Health-related quality of life (HRQoL), defined as overall physical, mental, and social well-being, tends to be higher among individuals with greater nutrition knowledge and healthier lifestyles, which is also associated with improved medication adherence (Suhail *et al*., 2021).

Hypertension is a significant public health concern in Ghana, affecting over one in four adults, with a prevalence ranging from 27% to 30.3% across both rural and urban areas (Atibila *et al*., 2021). The Northern and Upper East regions are characterized by an agrarian lifestyle that traditionally provides high levels of physical activity through subsistence farming. While the diet is historically centred on fibre-rich staples like ‘*tuo zaafi’* (a local traditional food). Due to recent modernization and convenience, these societies are undergoing a significant shift towards processed foods high in refined carbohydrates, salt, and unhealthy fats, with widespread use of bouillon cubes. Culturally, alcohol consumption from local brews like *pito* and high salt intake are prevalent. This epidemiological transition from active, plant-based living to more urbanized patterns with increased dietary risks is strongly linked to rising hypertension, compounded by low awareness and control of the condition (Sanuade *et al*., 2018). Besides, the Central and Western regions feature a coastal economy with fishing, farming, and growing urban service sectors, leading to a mix of physically active and increasingly sedentary lifestyles. The traditional diet relies on starchy staples like “fufu*”* and “kenkey”, which is now characterized by high consumption of fried foods, salted or smoked fish, palm oil, and processed snacks, often purchased from street vendors (Addo *et al*., 2007). This shift to an energy-dense, high-sodium diet is compounded by significant alcohol intake, particularly of local spirits like “akpeteshie”, and urban stressors. Together, these factors create a distinctly high-risk, obesogenic environment strongly associated with hypertension and metabolic disorders in these regions (Lie *et al*., 2018). Yet, awareness and management remain low, with only 35% aware of their condition, 22% receiving treatment, and just 6% having their BP under control (Atibila *et al.*, 2021). Ogedegbe *et al*. (2014) reported hypertension rates from 19.3% in rural areas to 54.6% in urban settings, with similarly low levels of detection, treatment, and control. A population-based study echoed these findings, showing prevalence rates between 19% and 48%, alongside poor awareness and management. The Ghana Health Service (GHS) reported in 2007 that hypertension is the leading cause of death in the country (Obirikorang *et al*., 2018). In the Central Region, prevalence rose from 2.1% in 2009 to 4.1% in 2012, with Cape Coast reaching 18.2% (Der and Marie-Antoinette, 2017). Studies also revealed that, the prevalence of hypertension ranged from 19% to 48% in rural and urban areas, respectively (Appiah *et al*., 2021; Bosu and Bosu, 2021). According to other research, significant population growth, an increase in life expectancy, and factors related to lifestyle have all been linked to hypertension (Cappuccio *et al*., 2006), which is now Ghana’s fifth most common cause of outpatient morbidity (de-Graft Aikins *et al*., 2014). The Ghana Ministry of Health indicated that, approximately half of all individuals currently have hypertension, up from less than 5% a generation ago (MOH, 2012). Scholars have remarked that, residing in the Central and Western regions increased the chance to having hypertension (Appiah *et al.*, 2021), because the proximity of these regions to the sea and availability of sea food could suggest that there is a higher consumption of salted fish and other sea food which are fortified with sodium (Khan *et al*., 2011). Conversely, the Upper East and Northern region also has limited access routes to health care and community facilities, hence has necessitated their inclusion in this study. The goal of lifestyle medicine is to empower individuals to take control of their own health by adopting healthy lifestyle habits, which in turn can help reduce the burden of chronic diseases, particularly on hypertension. Despite increasing rates in the Upper East, Northern, Central, and Western regions, there remains limited understanding of how lifestyle medicine and behavioural approaches such as healthy dietary adherence influence hypertension control. Therefore, this study aims to assess demographic factors, knowledge, and lifestyle behaviours related to hypertension management in urban populations across these four regions in Ghana.

## Methods

### Study design and setting

A cross-sectional analytical survey was conducted in four urban areas across four regions (Upper East, Northern, Central, and Western regions) in Ghana. The urban areas were located in Bolgatanga (Upper East), Tamale (Northern Region), Cape Coast (Central Region), and Effia Nkwanta (Western Region). The target population for the study included all participants aged 18–67^+^ years. The four regions (Upper East, Northern, Central, and Western) were purposively selected to capture populations with specific risk factors and access profiles relevant to hypertension. The Central and Western regions were included due to evidence suggesting elevated hypertension risk linked to dietary patterns, particularly higher consumption of sodium-rich preserved seafood (Appiah *et al.*, 2021; Khan *et al.*, 2011). Conversely, the Upper East and Northern regions were selected due to their documented limited access to healthcare infrastructure and community health resources, which impacts health literacy and disease management. Together, these regions represent distinct but important contexts; diet-driven risk and access-driven risk for studying how knowledge of lifestyle practices influences hypertension rates, as compared to the better-resourced capital.

### Study sites characteristics

Ghana, located in West Africa, covers an area of 238,533 square kilometres (92,099 square miles). It shares borders with Togo to the east, Côte d’Ivoire to the west, Burkina Faso to the north, and the Gulf of Guinea to the south. Ghana is politically and administratively divided into 16 regions and 260 districts. With a population of 30,792,608, of which 50.7% are female (Ghana Statistical Service, 2021), Ghana is the second most populous country in West Africa. Accra serves as both the capital and the largest city. The population of hypertensive patients in Ghana’s Upper East, Northern, Central, and Western regions vary, with regional differences in prevalence. Nationwide, the prevalence of hypertension is estimated to range from 27% to 30% of the adult population.

### Participants

**Figure 1.**
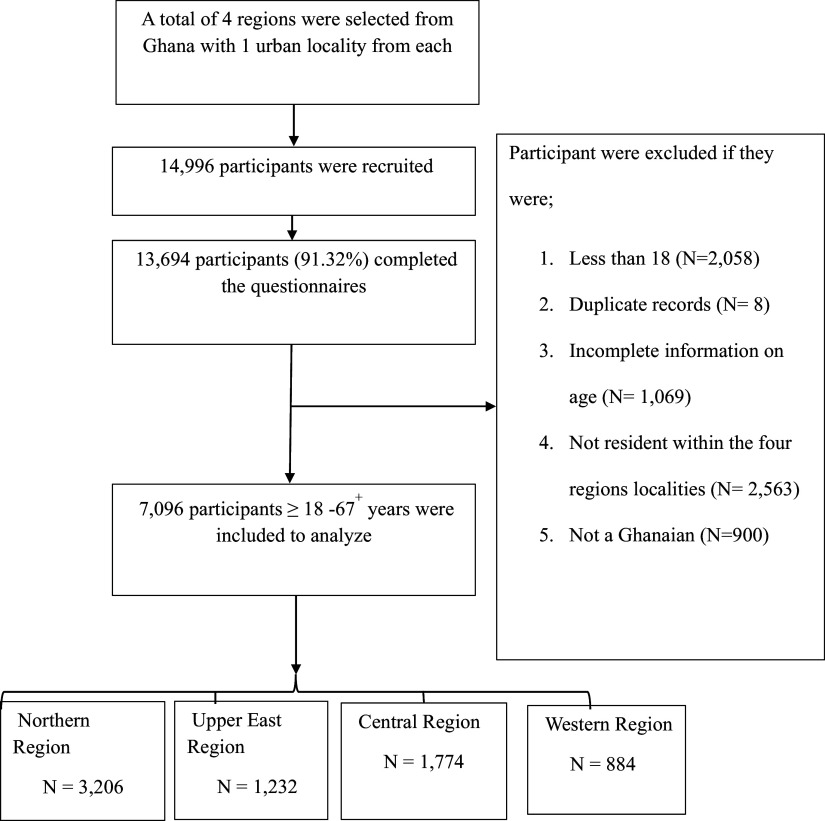
Flowchart of participant selection.

A total of 14,996 participants were interviewed across the four regions in this study from August 2023 and September 2024. 7096 valid participant data were included in the final analysis from the four regions. Invalid participants were removed based on the inclusion and exclusion criteria (Figure 1).

### Inclusion and exclusion criteria

To be considered for this study, the participant must be an adult aged 18–67^+^ years and a resident of Ghana for more than one year. This residency requirement was implemented to ensure participants had sufficient, stable exposure to the local environment and lifestyle factors relevant to the study’s objectives, thereby enhancing the sample’s representation of the Ghanaian population under investigation. The inclusion and exclusion criteria are provided in Figure 1. Participants diagnosed with hypertension and taking prescribed antihypertensive medication were included in the study, as the investigation of management behaviours was a core objective. Participants taking medications for non-hypertensive purposes known to directly and acutely affect blood pressure (e.g., systemic corticosteroids, stimulants) were excluded. Pregnant women were also excluded from the study. The total population was 14,996, of which 7900 was excluded due to missing data, including persons below 18 years, which resulted in 7096 as the analytical sample.

### Sampling

This study focused on Ghana, and the samples were stratified into four administrative regions (Northern, Upper West, Western and Central) and within an urban locality each, resulting in four (4) strata representation. The study employed a stratified sampling design at the first stage, dividing the target population into four key geographic strata (regions) with known limited health knowledge. Within each stratified region, data collection occurred at designated screening locations. At the point of enrollment, a convenience sampling approach based on temporal availability was used; all individuals who were present and available for screening during the data collectors’ visit were invited to participate. Data collection was conducted at central screening points (e.g., local health posts, community centers) within each urban locality of the selected regions. The sample size was allocated equally across the four strata (disproportional allocation), with the target of recruiting one-quarter of participants from each geographic region, irrespective of their differing population sizes. Trained researchers and recruiters using both the local language and English were used in the survey collection. The total number of participants from each region and the sample sizes are depicted in Figure 1.

### Data collection and measures

Participants involved in the survey were interviewed face-to-face using survey methods that required planning and coordination [paper and pencil interview (PAPI)] according to (Dai *et al*., 2022). All study procedures were conducted ethically per the code of ethics of the world medical association (declaration of Helsinki), and the study was approved by the Institutional Review Board of the College of Public Health, Zhengzhou University, China and GHS Ethics Review Committee. A formal permission was secured from opinion leaders from all the four strata communities prior to the commencement of the study. A statement of the purpose and nature of the study was provided to all prospective participants, and they were assured that participation was voluntary. Subjects were asked to sign informed consent before participating in the study. The consent form emphasized that participants’ responses to all questions would be kept confidential. Participants were also given the option to refuse to answer any or all questions that they felt were too personal. The participation rates were high in all the sites ranging from 82% to 99%. Data was collected using a stratified random sampling. Out of the four strata, respondents were sampled out conveniently until the needed sample size was achieved. Four distinct sections made up the organized questionnaire that was given by the skilled interviewers in both the local tongue and English. The researchers (consisting of voluntary nurses and midwifes) with professional training in BP measurement, were told to support the participant’s arm, choose the appropriate cuff size, position it over a bare arm, and rest the individual for five minutes before the measurement. Blood pressure (BP) readings were taken with an Omron M5-I monitor using appropriate cuff sizes. A normal (standard) cuff was used, which is designed to fit upper arm circumferences ranging from 22 to 32 cm (equivalent to approximately 8.7–12.6 inches), were reviewed by two of the authors to ensure accuracy. The mean of the three readings was used for analysis following the methods of Agyemang et.al (Agyemang *et al*., 2006). According to the WHO (Organization, 1996), BP categories are defined as follows: Normal BP indicates SBP below 120 mmHg and DBP below 80 mmHg. Prehypertension is identified by a SBP range of 120–139 mmHg and/or diastolic of 80–89 mmHg. Stage I Hypertension is defined as SBP of 140–159 mmHg and/or DBP of 90–99 mmHg, while Stage II Hypertension is characterized by SBP of 160 mmHg or higher and/or DBP of 100 mmHg or higher. Participants were categorized as having controlled blood pressure based on this threshold. Individuals classified as having hypertension met the criteria for diagnosed Stage 1 hypertension, while those classified as having severe hypertension met the criteria for diagnosed Stage 2 hypertension according to the WHO standard.

The second section consisted of questions on the socio-demographics of participants, including age in single years, sex of participant, level of education, employment status, income and area of residence. The third section examined participants’ knowledge about hypertension, its causes, complications, comorbidities, treatment and control (lifestyle behaviours). Data on current medication use, including antihypertensive drugs, was collected via self-report. ‘Knowledge about hypertension’ was defined as an individual’s awareness and understanding of the condition, including what hypertension is and how it is diagnosed. The ‘Risk factors’ was defined as factors that contribute to hypertension development (such as obesity, smoking, excess salt intake, alcohol consumption, physical inactivity, stress, family history, and ageing). ‘Symptoms and complications’ were defined as awareness that hypertension is often asymptomatic but can result in serious complications such as stroke, heart attack, kidney failure, and vision loss if left uncontrolled. ‘Treatment and control’ were defined as the proportion of participants who were aware of and adhered to preventive and management measures for hypertension, including the adoption of a low-salt diet, regular physical activity, maintenance of a healthy body weight, reduction in alcohol consumption and tobacco use, as well as the recognition of the importance of routine BP monitoring, compliance with prescribed antihypertensive medication, and regular medical follow-up for effective long-term control. A final knowledge score was derived for each respondent by summing their responses, assessed on a 5-point Likert scale (1 = strongly disagree to 5 = strongly agree), across three core topics: (1) causes of hypertension, (2) complications of hypertension, and (3) treatment or managements. The internal consistency of this composite scale was acceptable (Cronbach’s *α* = 0.90). For interpretation, the total sum scores were categorized as: Low Knowledge (total score ≤ 9, mean item score ≤ 3), Moderate Knowledge (total score 10–11, mean item score > 3 and < 4), and High Knowledge (total score ≥ 12, mean item score ≥ 4). Consequently, lower total scores indicate lower overall knowledge, while higher scores reflect greater knowledge (Koopman *et al*., 2002). The higher the average score, the better performance participants make in the corresponding scale. Missing items or non-response were managed according to the scoring instructions provided by the tool. The WHO STEPS questionnaire was used as the survey instrument with slight modification, which has demonstrated reliability and convergent validity and takes about 20 min to complete.

### Data analysis

Frequencies and proportions of the background factors by category were calculated to describe the sample and illustrate its distribution. The means of continuous outcomes were estimated. The t-test was used to compare the means of the variables age and BMI. Cross-tabulations of participants’ sociodemographic and lifestyle factors influencing hypertension control were undertaken. The hypertension control status of each participant was determined by calculating the average of all SBP and DBP readings recorded. Blood pressure readings of <140/90 mmHg were classified as optimal control. All estimates and 95% confidence intervals were computed using survey-design procedures to account for the multi-stage sampling and sampling weights. Logistic regression models used survey-adjusted standard errors to produce odds ratios (ORs) and 95% CIs. All analyses were conducted in SPSS 27, and statistical significance was set at a 95% confidence level.

## Results

### Sociodemographic characteristics and regional distribution of the study participants

The study involved 7,096 participants with a mean age of 37.27 ± 8.73 years. A comprehensive analysis revealed significant demographic and lifestyle variations across the four Ghanaian regions, as confirmed by chi-square tests. A highly significant association was found between region and gender (*p* < 0.001), with the Upper East having the highest male proportion (37.31%, 95% CI: 35.43–39.23) and the Western region the lowest (10.64%, 95% CI: 9.49–11.89). Age distribution also varied (*p* < 0.001); the Central and Western regions were dominated by younger adults (27–36 years), while the Northern and Upper East regions had older profiles. In contrast, no significant association was found for marital status (*p* = 0.100), as the vast majority in all regions were married. Educational attainment was significantly linked to region (*p* < 0.001), with the Western region having the highest rate of no formal schooling (36.21%, 95% CI: 33.44–39.07). The Western region was a consistent outlier in lifestyle factors, demonstrating near-universal abstinence from smoking (95.96% never smoked, 95% CI: 94.61–97.02) and drinking (98.92% never drank, 95% CI: 98.14–99.38), associations that were both highly significant (*p* < 0.001). While most individuals across all regions had a BMI in the healthy range, a significant association existed (*p* < 0.001), with the Western region having a slightly higher overweight rate. Finally, a very strong significant association was found with income (*p* < 0.001), characterized by a high proportion of low-income individuals in the Northern region and a more polarized distribution in the Western region (Table 1).

**Table 1.** Sociodemographic characteristics and regional distribution of the study participants

Variables	Central *N* (%) [95% CI]	Northern *N* (%) [95% CI]	Upper East *N* (%) [95% CI]	Western *N* (%) [95% CI]	*χ*^2^	*p*-Value
**Gender**						
Male	516 (20.12) [18.59–21.73]	819 (31.93) [30.10–33.82]	957 (37.31) [35.43–39.23]	273 (10.64) [9.49–11.89]	102.53	<0.001
Female	774 (17.08) [16.02–18.19]	1555 (34.32) [32.93–35.74]	1362 (30.06) [28.77–31.38]	840 (18.54) [17.44–19.69]		
**Age (Yrs)**						
18–26	123 (9.53) [8.02–11.27]	348 (14.66) [13.26–16.17]	279 (12.03) [10.78–13.39]	98 (8.81) [7.24–10.65]	583.41	<0.001
27–36	577 (44.73) [42.00–47.48]	521 (21.95) [20.33–23.65]	756 (32.60) [30.76–34.50]	616 (55.35) [52.42–58.25]		
37–46	279 (21.63) [19.46–23.96]	902 (37.99) [36.09–39.93]	852 (36.74) [34.85–38.67]	250 (22.46) [20.08–25.03]		
47–56	212 (16.43) [14.48–18.57]	538 (22.66) [20.99–24.42]	356 (15.35) [13.93–16.88]	111 (9.97) [8.30–11.92]		
57–66	96 (7.44) [6.10–9.02]	61 (2.57) [2.00–3.28]	65 (2.80) [2.20–3.54]	36 (3.23) [2.33–4.44]		
67^+^	3 (0.23) [0.08–0.67]	4 (0.17) [0.06–0.43]	11 (0.47) [0.26–0.84]	2 (0.18) [0.05–0.65]		
**Marital status**						
Single	6 (0.47) [0.21–1.01]	3 (0.13) [0.04–0.38]	12 (0.52) [0.29–0.90]	3 (0.27) [0.09–0.78]	14.67	0.100
Married	1263 (97.91) [96.90–98.61]	2326 (97.98) [97.38–98.44]	2246 (96.85) [96.06–97.50]	1095 (98.38) [97.42–99.01]		
Divorced	1 (0.08) [0.01–0.44]	3 (0.13) [0.04–0.38]	5 (0.22) [0.09–0.51]	1 (0.09) [0.02–0.50]		
Widowed	20 (1.55) [0.99–2.40]	42 (1.77) [1.31–2.38]	56 (2.41) [1.86–3.12]	14 (1.26) [0.74–2.11]		
**Educational level**						
Never attended	347 (26.90) [24.56–29.35]	680 (28.64) [26.83–30.53]	627 (27.04) [25.25–28.90]	403 (36.21) [33.44–39.07]	55.31	<0.001
Primary	425 (32.95) [30.45–35.54]	858 (36.14) [34.23–38.10]	773 (33.33) [31.47–35.26]	374 (33.60) [30.89–36.41]		
Secondary	505 (39.15) [36.52–41.84]	821 (34.58) [32.70–36.51]	894 (38.55) [36.64–40.50]	325 (29.20) [26.63–31.90]		
Tertiary	13 (1.01) [0.58–1.71]	15 (0.63) [0.38–1.04]	25 (1.08) [0.72–1.59]	11 (0.99) [0.55–1.76]		
**Smoking status**						
Never smoke	978 (75.81) [73.40–78.06]	1890 (79.61) [77.93–81.17]	1707 (73.61) [71.78–75.36]	1068 (95.96) [94.61–97.02]	252.38	<0.001
Still smoking	270 (20.93) [18.80–23.22]	443 (18.66) [17.12–20.30]	545 (23.50) [21.78–25.30]	34 (3.05) [2.18–4.25]		
Quit smoking	42 (3.26) [2.41–4.38]	41 (1.73) [1.27–2.33]	67 (2.89) [2.28–3.64]	11 (0.99) [0.55–1.76]		
**Drinking status**						
Never drinking	1212 (93.95) [92.52–95.12]	2271 (95.66) [94.73–96.43]	2125 (91.63) [90.45–92.70]	1101 (98.92) [98.14–99.38]	86.07	<0.001
Drinking	78 (6.05) [4.88–7.48]	103 (4.34) [3.59–5.22]	194 (8.37) [7.29–9.57]	12 (1.08) [0.61–1.88]		
**BMI**						
<25	887 (68.76) [66.17–71.24]	1614 (67.99) [66.09–69.83]	1619 (69.81) [67.93–71.62]	741 (66.58) [63.77–69.28]	265.42	<0.001
25–29.9	365 (28.29) [25.87–30.83]	685 (28.85) [27.08–30.69]	625 (26.95) [25.17–28.80]	342 (30.73) [28.09–33.48]		
≥30	39 (3.02) [2.21–4.11]	75 (3.16) [2.53–3.93]	75 (3.23) [2.58–4.03]	30 (2.70) [1.88–3.84]		
**Monthly income (GH¢)**						
Less than 1000	90 (20.45) [16.87–24.53]	180 (40.91) [36.31–45.64]	150 (34.09) [29.67–38.78]	20 (4.55) [2.94–6.95]	683.51	<0.001
1000–5000	600 (11.62) [10.77–12.52]	2514 (48.67) [47.24–50.10]	1932 (37.41) [36.07–38.77]	119 (2.30) [1.93–2.74]		
5000 and above	313 (17.21) [15.55–19.00]	643 (35.35) [33.21–37.55]	614 (33.75) [31.64–35.93]	273 (15.01) [13.46–16.68]		

### Prevalence of hypertension by age, sex, and region

**Figure 2.**
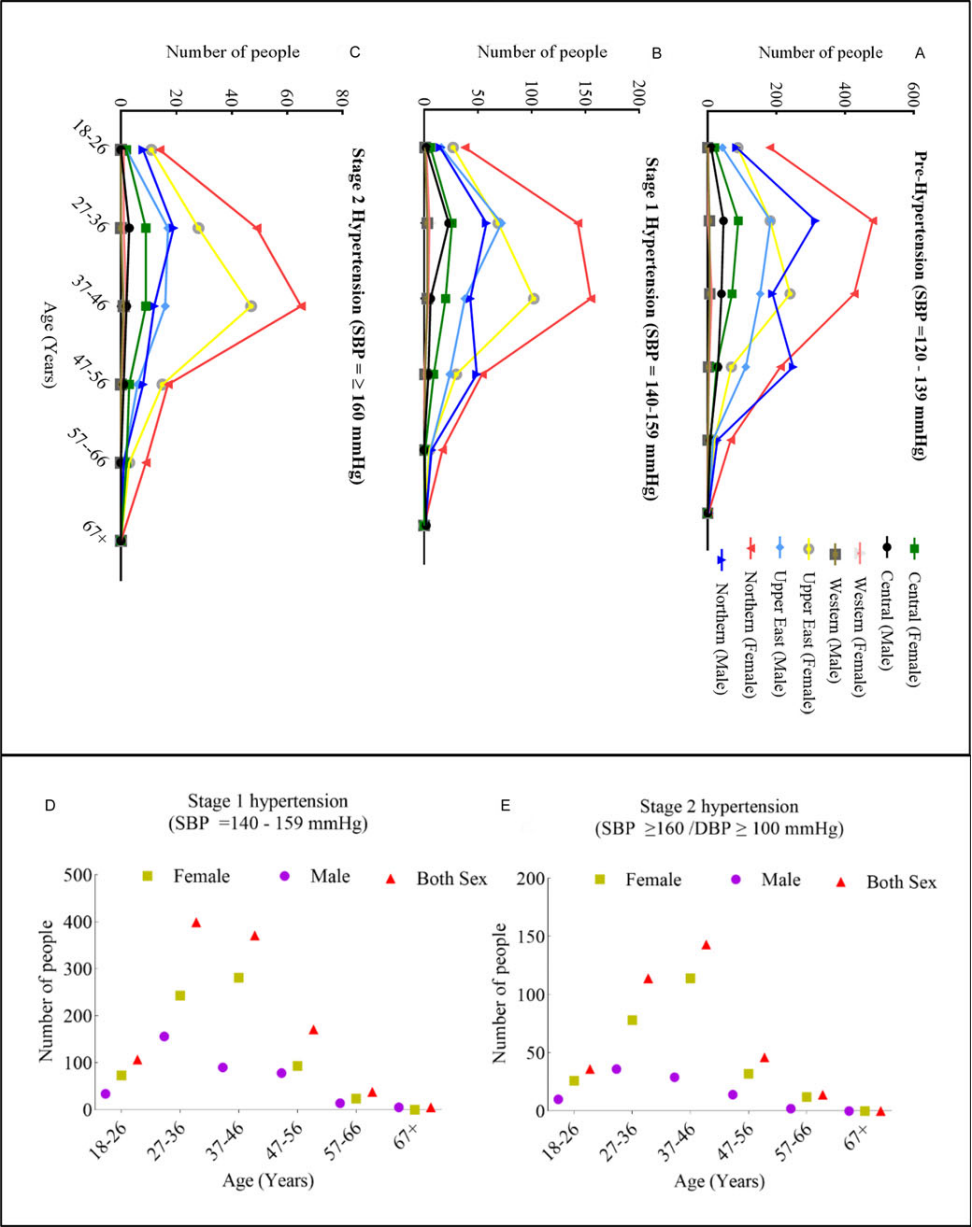
Prevalence of people living with hypertension by age, sex and region. Where **A.** Pre-hypertension and **B.** Stage 1-hypertension, **C**. Stage 2-hypertension, **D**. Overall number of people living with stage 1 and **E**. Overall number of people living with stage 2 hypertension.

The prevalence of pre-hypertension and hypertension varied significantly across age, sex, and region in urban Ghana, revealing a clear geographic gradient. The Northern and Upper East regions consistently bore the highest burden, with the Northern region recording the highest proportions of both Stage 1 (50.30%) and Stage 2 (52.6%) hypertension, while the Upper East region had the highest rate of pre-hypertension. A strong sex disparity was evident. Females consistently constituted the majority of hypertensive cases across most age groups and regions, particularly for Stage 2 hypertension (Figure 2A–C). However, the highest Stage 2 prevalence occurred in the 37–46-year group.

Overall, among the 1091 individuals diagnosed with Stage 1 hypertension, females constituted 63%, while males accounted for 37%, indicating a notable gender disparity. The highest prevalence was observed in the 27–36-year age group (37%), followed closely by the 37–46-year age group (34%). In contrast, the lowest prevalence was recorded in the 67^+^ age group (*p* < 0.5%), largely attributable to the limited number of participants within this demographic (Figure 2D–E)). Of the 349 individuals identified with Stage 2 hypertension, a pronounced gender imbalance was also evident, with females representing 71% (*n* = 248) of cases compared to 29% (*n* = 101) among males.

### Multivariate analysis of socio-demographic factors on high blood pressure

In the fully adjusted models, both systolic (OR = 1.02, CI: 1.01–1.03) and DBP (OR = 1.06, CI: 1.05–1.07) were independently associated with increased odds of hypertension. Several behavioural and lifestyle factors demonstrated significant associations. Adjusting for key sociodemographic factors, revealed notable associations with hypertension diagnosis. Counterintuitively, the regression analysis indicated that both smoking (OR = 0.43, CI: 0.22–0.86) and self-reported excessive salt intake (OR = 0.48, CI: 0.30–0.76) were associated with significantly lower odds of being diagnosed with hypertension. Conversely, excessive alcohol intake (OR = 1.29, CI: 1.01–1.66), awareness of hypertension as a ‘silent killer’ (OR = 1.78, CI: 1.13–2.80), and a history of alcohol cessation (OR = 1.35, CI: 1.04–1.75) were associated with higher odds of hypertension diagnosis. Regarding physical activity, our results show that regular exercise (OR = 0.72, CI: 0.54–0.96) was inversely associated with hypertension, indicating that participants with higher engagement in physical activity had lower odds of being hypertensive.

Knowledge about risk factors and consequences of hypertension, kidney damage (OR = 0.58, CI: 0.34–0.99,), stroke (OR = 0.59, CI: 0.37–0.93,), vision problems (OR = 0.51, CI: 0.27–0.96), and mortality (OR = 0.48, CI: 0.33–0.70) were significantly associated with reduced odds. Similarly, knowledge of treatment and prevention measures such as lifestyle changes (OR = 0.48, CI: 0.28–0.82), weight loss (OR = 0.63, CI: 0.39–0.99), and dietary control (OR = 0.33, CI: 0.15–0.73, *p*<0.05) were protective against hypertension (Table 2).

**Table 2.** Multivariate logistic regression of knowledge, lifestyle behaviour and social support factors associated with hypertension control

Category of variables	Model 1^a^ OR (95% CI)	Model 2^b^ OR (95% CI)	Model 3^c^ OR (95% CI)	Model 4^d^ OR (95% CI)
Systolic blood pressure	1.04 (1.01–1.07)*	1.v02 (1.01–1.03)*	1.02 (1.01–1.03)*	1.02 (1.01–1.03)*
Diastolic blood pressure	1.02 (1.01–1.03)*	1.02 (1.01–1.03)*	1.06 (1.05–1.07)***	1.06 (1.05–1.07)***
**Knowledge about hypertension causes and symptoms**				
Prehypertension → Hypertension	0.63 (0.41–0.98)*	0.63 (0.41–0.99)*	0.65 (0.41–1.02)	0.64 (0.40–1.02)
Smoking	0.46 (0.24–0.88)*	0.43 (0.23–0.83)*	0.44 (0.22–0.85)*	0.43 (0.22–0.86)*
Excessive salt intake	0.57 (0.37–0.87)*	0.58 (0.37–0.89)*	0.51 (0.32–0.79)*	0.48 (0.30–0.76)*
Excessive alcohol intake	1.22 (0.97–1.56)	1.26 (0.99–1.61)	1.31 (1.03–1.67)*	1.29 (1.01–1.66)*
Stress	1.21 (0.57–2.50)	1.22 (0.57–2.60)	1.19 (0.55–2.58)	1.16 (0.53–2.55)
Lack of physical activity	1.08 (0.81–1.45)	1.05 (0.81–1.38)	1.09 (0.83–1.43)	1.82 (0.73–4.59)
Silent killer awareness	1.63 (1.17–2.27)*	1.59 (1.14–2.23)*	1.65 (1.17–2.31)*	1.78 (1.13–2.80)*
Sleeplessness	0.59 (0.32–1.03)	0.58 (0.32–1.03)	0.56 (0.30–1.03)	0.55 (0.29–1.03)
**Knowledge about hypertension, risk factors and consequences**				
Genetic	0.93 (0.76–1.14)	0.95 (0.79–1.14)	0.97 (0.80–1.18)	0.98 (0.49–1.94)
Age	1.07 (0.86–1.32)	1.10 (0.88–1.38)	1.17 (0.66–2.07)	1.20 (0.67–2.16)
Kidney damage	0.69 (0.42–1.16)	0.68 (0.40–1.14)	0.65 (0.38–1.10)	0.58 (0.34–0.99)*
Stroke	0.59 (0.38–0.91)*	0.58 (0.37–0.90)*	0.58 (0.37–0.92)*	0.59 (0.37–0.93)*
Cholesterol	1.18 (0.77–1.81)	1.22 (0.79–1.89)	1.24 (0.80–1.93)	1.25 (0.79–1.97)
Vision problems	0.54 (0.30–0.98)*	0.56 (0.30–1.02)	0.55 (0.30–1.02)	0.51 (0.27–0.96)*
Mortality	0.57 (0.40–0.82)*	0.56 (0.39–0.81)*	0.53 (0.37–0.77)*	0.48 (0.33–0.70)*
**Knowledge about hypertension treatment and prevention**				
Lifestyle changes	0.53 (0.32–0.88)*	0.54 (0.32–0.90)*	0.56 (0.33–0.93)*	0.48 (0.28–0.82)*
Weight loss	0.60 (0.38–0.93)*	0.59 (0.38–0.92)*	0.59 (0.38–0.93)*	0.63 (0.39–0.99)*
Dietary control	0.34 (0.16–0.71)*	0.34 (0.16–0.74)*	0.37 (0.17–0.80)*	0.33 (0.15–0.73)*
Alcohol cessation	1.27 (1.00–1.63)*	1.31 (1.02–1.67)*	1.36 (1.05–1.74)*	1.35 (1.04–1.75)*
Regular exercise	0.72 (0.50–0.99)*	0.72 (0.50–0.99)*	0.72 (0.50–0.99)*	0.72 (0.50–0.99)*
**Provision of social support related to lifestyle modification**				
Health education importance	0.88 (0.76–1.01)	0.88 (0.77–1.01)	0.87 (0.75–1.00)	0.89 (0.77–1.03)
Access to peer support groups	1.11 (1.01–1.21)*	1.11 (1.01–1.21)*	1.11 (1.02–1.22)*	1.11 (1.01–1.22)*
Limit salt/processed food	0.85 (0.75–0.96)*	0.85 (0.75–0.97)*	0.84 (0.74–0.96)*	0.88 (0.78–1.01)
Family support for regular exercise	0.88 (0.80–0.95)*	0.88 (0.80–0.97)*	0.87 (0.79–0.97)*	0.86 (0.77–0.95)*

*p < 0.1; ***p < 0.01; reference group was controlled hypertension; ^a^model 1 was adjusted for age and gender; ^b^model 2 was adjusted for age, gender, and marital status; ^c^model 3 was adjusted for age, gender, marital status, level of education, body mass index, and annual income; ^d^model 4 was adjusted for age, gender, marital status, level of education, body mass index, annual income, smoking, and alcohol consumption. OR<1 indicates a protective association (lower odds of hypertension), while OR>1 indicates increased odds.

Social support and workplace factors also influenced hypertension risk (*p* < 0.05). Access to peer support groups was positively associated with hypertension (OR = 1.11, CI: 1.01–1.22), while limitation of salt and processed food intake (OR = 0.84, CI: 0.74–0.96) and family support for regular exercise (OR = 0.86, CI: 0.77–0.95) lowers the odds of being diagnosed, suggesting the importance of supportive environments in promoting healthy behaviours. These findings suggest that both knowledge and practical adherence to lifestyle recommendations are important determinants of hypertension risk. Although behaviours established as biological risk factors (smoking, excessive salt intake) were associated with a lower likelihood of hypertension diagnosis, while health awareness was associated with a higher likelihood, high BP persists in some participants despite awareness, emphasizing the need for multifaceted interventions combining education with sustained behaviour change.

### Associations of demographic factors and lifestyle barriers to hypertension management

Older age was the strongest demographic predictor, with the likelihood of encountering barriers to hypertension management increasing substantially with each successive age group (*p* < 0.001). The risk was highest among adults aged 67 and older. While initial analysis suggested women faced greater barriers, adjusted results indicated that, when accounting for other factors, women actually had a significantly lower likelihood of facing these challenges compared to men (CI: 0.47–0.67, *p* < 0.001). Marital status was significantly associated with barriers, as married participants were much more likely to report difficulties in managing hypertension (CI: 3.97–38.09, *p* < 0.001). Regarding lifestyle, those who are still smoking (CI: 2.01–3.92, *p* < 0.001) and those who quit smoking (CI: 1.38–2.14, *p* < 0.001) were strongly associated with a higher probability of encountering management barriers. A similar pattern was observed with alcohol use, where both current drinkers (CI: 1.38–2.76, *p* < 0.001) and those who had quit (CI: 1.12–1.85, *p* < 0.003) faced greater odds than lifetime abstainers (Table 3).

**Table 3.** Associations of demographic factors and lifestyle barriers to hypertension management (*n* = 7096)

Covariates	OR (95% CI)	*p*-Value	AOR (95% CI)	*p*-Value
**Age**				
18–26	ref	ref	ref	ref
27–36	2.63 (2.23–3.08)	<0.001	3.09 (2.59–3.68)	<0.001
37–46	1.49 (1.27–1.75)	<0.001	1.50 (1.26–1.77)	<0.001
47–56	5.17 (4.21–6.34)	<0.001	6.14 (4.90–7.68)	<0.001
57–66	9.43 (6.07–14.66)	<0.001	14.11 (8.61–23.12)	<0.001
67+	8.71 (5.01–15.12)	<0.001	18.91 (10.50–34.07)	<0.001
**Gender**				
Male	ref	ref	ref	ref
Female	1.36 (1.23–1.51)	<0.001	0.56 (0.47–0.67)	<0.001
**Marital status**				
Single	ref	ref	ref	ref
Married	11.88 (4.05–34.79)	<0.001	12.30 (3.97–38.09)	<0.001
Widowed	0.69 (0.21–2.28)	0.52	0.57 (0.16–2.00)	0.38
**Educational levels**				
Never attended	ref	ref	ref	ref
Primary	1.59 (0.95–2.66)	0.08	0.89 (0.77–1.03)	0.13
Secondary	1.50 (0.90–2.51)	0.11	0.65 (0.56–0.76)	<0.001
Tertiary	1.07 (0.64–1.78)	0.80	0.72 (0.39–1.30)	0.28
**Smoking status**				
Never smoker	ref	ref	ref	ref
Quit smoking	1.85 (1.55–2.21)	<0.001	1.72 (1.38–2.14)	<0.001
Still smoking	2.96 (2.20–3.97)	<0.001	2.81 (2.01–3.92)	<0.001
**Drinking status**				
Never drinker	ref	ref	ref	ref
Quit drink	1.61 (1.33–1.95)	<0.001	1.44 (1.12–1.85)	0.003
Current drinking	2.10 (1.58–2.79)	<0.001	1.95 (1.38–2.76)	<0.001
**Consumption of vegetables**				
Every day	ref	ref	ref	ref
Often	1.41 (1.20–1.67)	<0.001	1.35 (1.13–1.63)	0.001
Occasionally	1.20 (0.72–2.00)	0.48	1.13 (0.65–1.95)	0.67
**Lifestyle barriers**				
**Poverty**				
Strongly disagree	ref	ref	ref	ref
Do not agree	1.12 (0.74–1.69)	0.59	1.09 (0.68–1.75)	0.71
Generally	1.05 (0.70–1.57)	0.81	1.00 (0.63–1.58)	0.99
Agree	1.01 (0.67–1.52)	0.95	0.96 (0.61–1.50)	0.85
Totally agree	0.97 (0.63–1.48)	0.89	0.92 (0.57–1.48)	0.72
**Lack of time for physical activity**				
Strongly disagree	ref	ref	ref	ref
Do not agree	1.19 (0.85–1.68)	0.29	1.10 (0.74–1.64)	0.62
Generally	1.07 (0.76–1.51)	0.69	1.02 (0.68–1.54)	0.91
Agree	1.11 (0.84–1.46)	0.47	1.05 (0.72–1.51)	0.80
Totally agree	1.08 (0.80–1.46)	0.61	1.02 (0.69–1.50)	0.93
**Work schedule**				
Strongly disagree	ref	ref	ref	ref
Do not agree	1.05 (0.81–1.36)	0.68	1.08 (0.78–1.49)	0.63
Generally	1.09 (0.85–1.39)	0.51	1.06 (0.75–1.50)	0.74
Agree	1.01 (0.77–1.34)	0.94	1.00 (0.70–1.44)	0.99
Totally agree	0.95 (0.70–1.29)	0.75	0.97 (0.65–1.46)	0.88
**Social influences on alcohol/smoking**				
Strongly disagree	ref	ref	ref	ref
Do not agree	1.10 (0.95–1.28)	0.20	0.95 (0.70–1.30)	0.76
Generally	1.09 (0.95–1.26)	0.20	0.95 (0.69–1.30)	0.74
Agree	1.16 (0.91–1.48)	0.21	1.09 (0.73–1.61)	0.65
Totally agree	0.74 (0.53–1.05)	0.09	1.21 (0.74–1.98)	0.45
**Limited access to healthcare**				
Strongly disagree	ref	ref	ref	ref
Do not agree	1.05 (0.91–1.22)	0.51	1.11 (0.78–1.59)	0.55
Generally	1.06 (0.93–1.22)	0.34	1.17 (0.79–1.72)	0.43
Agree	1.16 (0.89–1.51)	0.27	1.10 (0.65–1.84)	0.72
Totally agree	0.71 (0.50–1.01)	0.06	0.94 (0.47–1.86)	0.85
**Lack of family/friends support**				
Strongly disagree	ref	ref	ref	ref
Do not agree	1.20 (0.85–1.68)	0.29	1.01 (0.74–1.39)	0.92
Generally	1.25 (0.90–1.73)	0.18	1.10 (0.78–1.55)	0.57
Agree	1.28 (0.90–1.81)	0.16	1.05 (0.72–1.54)	0.78
Totally agree	1.34 (0.95–1.89)	0.09	0.98 (0.65–1.49)	0.93
**Absence of recreational spaces**				
Strongly disagree	ref	ref	ref	ref
Do not agree	1.12 (0.74–1.70)	0.60	1.05 (0.67–1.65)	0.82
Generally	1.18 (0.81–1.72)	0.39	1.09 (0.71–1.67)	0.70
Agree	1.21 (0.83–1.77)	0.32	1.13 (0.73–1.73)	0.59
Totally agree	1.26 (0.87–1.82)	0.21	1.15 (0.75–1.77)	0.52
**Lack of knowledge about healthy lifestyle**				
Strongly disagree	ref	ref	ref	ref
Do not agree	1.24 (0.48–3.19)	0.66	1.14 (0.62–2.10)	0.67
Generally	1.32 (0.82–2.11)	0.25	1.20 (0.72–2.02)	0.49
Agree	1.38 (0.88–2.18)	0.16	1.25 (0.76–2.05)	0.38
Totally agree	1.41 (0.90–2.20)	0.13	1.28 (0.76–2.16)	0.35
**Cultural/traditional restriction**				
Strongly disagree	ref	ref	ref	ref
Do not agree	1.10 (0.73–1.66)	0.65	1.08 (0.69–1.70)	0.73
Generally	1.16 (0.78–1.74)	0.46	1.11 (0.71–1.74)	0.64
Agree	1.21 (0.81–1.80)	0.35	1.15 (0.73–1.81)	0.54
Totally agree	1.27 (0.86–1.88)	0.23	1.20 (0.77–1.87)	0.42
**Lack of stress management techniques**				
Strongly disagree (ref)	ref	ref	ref	ref
Do not agree	1.18 (0.77–1.80)	0.45	1.15 (0.73–1.82)	0.55
Generally	1.22 (0.81–1.84)	0.33	1.19 (0.76–1.86)	0.44
Agree	1.25 (0.84–1.87)	0.28	1.21 (0.77–1.91)	0.40
Totally agree	1.30 (0.88–1.93)	0.19	1.24 (0.79–1.96)	0.35

Values are expressed as Odds Ratios (OR) with 95% Confidence Intervals (CI). AOR = Adjusted Odds Ratio, derived from multivariate logistic regression including all listed covariates. ‘ref’ indicates the reference category for comparison. OR >1 indicates higher odds of reporting barriers relative to the reference, while OR<1 indicates lower odds.

### Correlation between sociodemographic factors and high blood pressure

Age demonstrated a moderate positive association with SBP (*r* = 0.35, *p* < 0.01) and DBP (*r* = 0.25, *p* < .05), consistent with the established trend of increasing BP with advancing age. BMI was strongly correlated with SBP (*r* = 0.60, *p* < 0.01) and moderately correlated with diastolic blood pressure (*r* = 0.35, *p* < 0.01), confirming its role as a major risk factor. Gender showed significant positive correlations with smoking (*r* = 0.40, *p* < 0.01) and drinking (*r* = 0.35, *p* < 0.01) and was weakly correlated with BP. Education was positively associated with income (*r* = 0.45, *p* < 0.01) but negatively correlated with smoking (*r* = –0.25, *p* < 0.05), drinking (*r* = –0.20, *p* < 0.05), and blood pressure. Smoking and drinking were positively but modestly associated with both SBP and DBP. In general, age, BMI, and lifestyle behaviours (smoking and drinking) emerged as key correlates of hypertension (Table 4).

**Table 4.** Correlation between sociodemographic factors and hypertension

Variables	Age	Gender	Marital status	Education	Annual income	Smoking status	Drinking status	BMI	Systolic BP	Diastolic BP
Age										
Gender	0.05									
Marital status	0.02	0.00								
Education	−0.10	−0.20	−0.05							
Annual income	0.05	0.10	0.02	0.45**						
Smoking status	0.05	0.40**	0.02	−0.25*	−0.05					
Drinking status	0.06	0.35**	0.03	−0.20*	0.05	0.30**				
BMI	0.10*	0.05	0.00	−0.08	0.08*	0.05	0.06			
Systolic BP	0.35**	0.12*	0.04	−0.10	0.02	0.10*	0.12*	0.60**		
Diastolic BP	0.25*	0.08*	0.03	−0.08	−0.01	0.08*	0.09*	0.35**	0.70**	

**Correlation is significant at the 0.01 level (2-tailed). *Correlation is significant at the 0.05 level (2-tailed). Where 1. Age, 2. Gender, 3. Marital status, 4. Level of education, 5. Annual income, 6. Smoking status, 7. Drinking status, 8. BMI, 9. Systolic blood pressure, 10. Diastolic blood pressure.

## Discussion

This study examined the knowledge, lifestyle behaviours, and socio-demographic factors associated with hypertension control among adults in Northern, Upper East, Central and Western regions of Ghana. The study involved 7096 participants with 36.1% of male’s participants, while females constitute approximately 63.9%. This sex variation of the study participant might have explained the inclusion of more females than males. Studies had been shown that the incidence and the progression rate of cardiovascular disease and hypertension is markedly higher in men than in age-matched, premenopausal women (Reckelhoff, 2001; Yadav *et al*., 2008). But after menopause, this relationship no longer exists, and in old age women have similar rates of cardiovascular disease, and even higher prevalence of hypertension than men (Mosca *et al*., 1997). The mean age of participants in this current study was 37.27 years. However regional distributions of both gender and age differed significantly. The Upper East region had the highest proportion of male participants (37.31%), while the Western region had the lowest (10.64%) with high number of females than males. Demographically, the Central and Western regions were dominated by younger adults (27–36 years), whereas the Northern and Upper East regions had older population profiles. This age structure of the study population might have explained the inclusion of young adults with more females than males.

The Third National Health and Nutrition Evaluation Survey (NHANES III) showed that the prevalence of hypertension increases with advancing age to the point where more than half of people 60–69 years of age and approximately three-fourths of those 70 years of age and older are affected (Burt *et al*., 1995). However, it is worth noting that although the current study used a convenience sample, it found that 63% of females and 37% of males had hypertension, as discussed above. The highest prevalence was observed in the 27–36-year age group (37%), followed closely by the 37–46-year age group (34%). Our findings are consistent with those of (Woodiwiss *et al*., 2025) who reported that 15.6% of young South African adults (<40 years of age) have hypertension, with awareness among those affected being remarkably low (24.0%). Similarly, a study of young adults in the United States found low awareness levels (32% in women and 25% in men with hypertension), despite a notable prevalence (12% in young women and 27% in young men) (Everett and Zajacova, 2015). These age discrepancies in awareness have been attributed to several factors; younger adults tend to be healthier, they have higher rates of smoking and alcohol intake, they consume more fatty foods leading to weight gain, and they are less likely to see doctors regularly. This combination decreases the likelihood that they will have accurate and up-to-date knowledge of their BP status.

However, there was an impact of locality or region on the prevalence of hypertension in the current study where the Northern and Upper East regions consistently bore the highest burden, with the Northern region recording the highest proportions of both Stage 1 (50.30%) and Stage 2 (52.6%) diagnosed hypertension, while the Upper East region had the highest rate of pre-hypertension. This finding was similar to those of (Dai *et al.*, 2022) who reported that there was a 53.72% hypertension prevalence rate among older adults of Ghana with stratified sample from ten administrative regions (Ashanti, Brong Ahafo, Central, Eastern, Greater Accra, Northern, Upper East, Upper West, Volta, and Western) and type of locality (urban/rural). The regional differences in the prevalence of hypertension in this current study could be attributed to gender disparity, low levels of hypertension awareness, cultural practices and low knowledge about hypertension. In addition, factors like changes in diet, lifestyle, and the rising prevalence of obesity may contribute to finding a higher prevalence of hypertension in these localities (Addo *et al*., 2012). The overall prevalence rate of hypertension reported in this study is closely similar to previous studies, indicating the need for concern. Hence, developing primary intervention strategies is vital to curb the prevalence of hypertension among both young and old adults in Ghana.

Lifestyle modification is a comprehensive approach that emphasizes the adoption of various healthy practices to enhance overall well-being, particularly for individuals with or at risk of hypertension. Findings from this study indicates that smoking and excessive salt intake were associated with lower odds of hypertension diagnosis. A primary explanation is that, individuals who smoke or consume high-salt diets but are not engaged with primary care may remain undiagnosed. Besides, knowledge about risk factors and consequences of hypertension such kidney damage, stroke, vision problems, and mortality in this current study was moderate. However, knowledge of specific treatment and prevention measures such as lifestyle changes, weight loss, and dietary control was poor. This finding aligns with prior research. For example, (Adjei *et al.*, 2024) reported that only 36% of participants in Ghana had good knowledge of lifestyle modifications for hypertension management. Similarly a study conducted in North-western Nigeria (Hadiza *et al.*, 2017) found that, just 31.7% of participants possessed such knowledge. These consistently low levels of awareness highlight an urgent need for targeted educational interventions on hypertension management through lifestyle modification in Ghana.

Our study confirms the role of several established cardio-metabolic risk factors in the urban Ghanaian context. The strong and moderate correlations observed between BMI, SBP, and DSP, respectively, underscore obesity as a paramount driver of hypertension, a finding consistent with global reports, e.g., (Dai *et al.*, 2022). Furthermore, excessive alcohol consumption maintained its expected association with increased hypertension risk in our model. These alignments with international evidence reinforce that the biological pathways of hypertension operate similarly in Ghana and validate the relevance of global prevention strategies targeting weight management and harmful alcohol use.

In the logistic regression analysis, both systolic and DBP were independently associated with increased odds of hypertension. Several behavioural and lifestyle factors demonstrated significant associations. Smoking and excessive salt intake were associated with lower hypertension risk. These counterintuitive associations are highly unlikely to reflect true biological effects, where smoking and high salt intake are known risk factors (Dorner *et al.*, 2012), but are best explained by systematic bias inherent to cross-sectional studies of diagnosed disease. The most compelling explanation is that, individuals who are more health-aware or who engage with the healthcare system for other reasons are more likely to be screened. This leads to a higher probability of diagnosis among the ‘aware’ group, explaining the positive association**.** Conversely, individuals who smoke or consume high-salt diets but do not seek regular medical care are more likely to have undiagnosed hypertension. Their over-representation in the non-hypertensive control group artificially creates protective ORs< 1. This creates a form of reverse causality, where diagnosis status influences the measurement of exposures and awareness. This framework also clarifies the association between awareness of hypertension as a ‘silent killer’ and higher diagnosis odds. This awareness is likely a consequence, not a cause, of healthcare engagement; patients are routinely counselled with this term following a diagnosis or risk assessment (Mills *et al.*, 2020). Also, the high level of awareness could be dependent on the importance attached to knowing one’s status which is somewhat associated with one’s level of education, since most of our study participants had secondary education. Therefore, these paradoxical results do not contradict the well-documented role of salt (He and MacGregor, 2002) or smoking (Virdis *et al.*, 2010) in hypertension pathophysiology, instead, they underscore a major public health reality. In urban Ghana, diagnosis is strongly correlated with healthcare access and prior health engagement, leaving a significant burden of undiagnosed hypertension among those who are less aware or less connected to clinical services.

Barriers to hypertension management in this study were strongly patterned by demographic and lifestyle factors. Age showed a striking gradient, with older adults reporting progressively higher barriers, consistent with previous research that multi-morbidity, fixed incomes, and reduced mobility complicate self-care (Holland *et al*., 2024; Nwadiugwu, 2021). van de Vijver *et al*., (2013) reported that targeting high risk individuals and groups may be an effective strategy to reduce barriers leading to hypertension. While crude odds suggested more barriers among women, adjustment odds reversed the association, reflecting women’s generally have greater health-seeking behaviour once age and marital status were accounted for. Married participants were more likely to report barriers than singles, possibly due to competing household demands and shared dietary environments. Secondary education was protective, highlighting the role of health literacy, while lifestyle behaviours (smoking, alcohol use) displayed a clear stepwise gradient. Specifically, never-users reported the fewest barriers, former users reported more, and current users the most, showing both withdrawal challenges and heightened awareness among quitters. In Ghana the proportion of men who smoke compared to women is very high, hence the smoking status in our study may have larger number of men than women. Our findings are in consistent with those of (Li *et al*., 2017), who reported that the prevalence of hypertension in men who are former smokers was high. The impact of quitting smoking on other variables that may have an impact on blood pressure must be taken into account when interpreting the change in BP following quitting smoking. Among these factors are a decrease in alcohol consumption and an increase in overall food intake, which leads to weight gain (Puddey *et al*., 1985). A similar trend was observed in the study of (Lee *et al*., 2001), and adjustment of these changes did not alter their relationship.

However, diet also plays an integral part in hypertension prevention. The main components of the dietary factor for hypertension prevention are increased fruit and vegetable consumption (Borgi *et al*., 2016). Likewise, this study corresponds and revealed that daily vegetable consumption among participants was associated with lower barriers, reinforcing diet quality as a proxy for food security and planning capacity. This may be explained by the fact that most Ghanaians consume vegetables frequently, particularly while preparing indigenous stews and soups. Meanwhile, structural barriers (poverty, time, access, cultural restrictions) showed no independent effect after adjustment, likely reflecting confounding with age and education. All things considered, our results highlight the necessity of behaviour-specific, literacy-focused, and age-sensitive treatments to lower barriers in the treatment of hypertension. From this study, awareness had statistically significant association with lifestyle modification. Participants who had high awareness on lifestyle modification recommended for hypertension management were more likely to manage the risks of hypertension compared to those who had low knowledge.

### Limitations of the study

Although this current study presents several advantages, however, there are some limitations. The use of convenience sampling method for the data collection has a methodological limitation. This presents the findings somehow bias because the convenience sampling target population that are easily accessible, geographical proximity, and willingness to participate at a given period of time. Secondly, key variables were based on self-report, which is susceptible to recall and social desirability biases. Thirdly, the study covered only four regions of Ghana which limits the generalizability of the findings. Future research should incorporate probability-based sampling across all regions, rural populations, and longitudinal designs to provide a more comprehensive understanding of hypertension in Ghana.

## Conclusion

This study concludes that while awareness of hypertension as a health threat exists among urban Ghanaians, its translation into effective prevention and management is critically hampered by systemic and behavioural gaps. The findings reveal a paradox; traditional risk factors like smoking and high salt intake were not associated with higher odds of diagnosed hypertension, while health awareness was linked to increased odds. This pattern does not indicate a protective effect of risk behaviours but rather exposes a significant burden of undiagnosed hypertension, likely concentrated among individuals less engaged with the healthcare system. Furthermore, the analysis confirmed that factors such as older age, being married, and high-risk behaviours like smoking present significant barriers to optimal hypertension control. Future interventions must be comprehensive, integrating continuous, practical education with structural support for behaviour change, tailored to high-risk demographic groups identified in this study, while actively involving community and family support systems to foster environments conducive to healthy living. Addressing these multifaceted challenges is essential to curbing the increasing burden of hypertension in Ghana.

## Data Availability

Data available on request due to privacy/ethical restriction.
